# Inhibition of BDNF in Multiple Myeloma Blocks Osteoclastogenesis via Down-Regulated Stroma-Derived RANKL Expression Both In Vitro and In Vivo

**DOI:** 10.1371/journal.pone.0046287

**Published:** 2012-10-15

**Authors:** Li-Sha Ai, Chun-Yan Sun, Lu Zhang, Shun-Chang Zhou, Zhang-Bo Chu, You Qin, Ya-Dan Wang, Wei Zeng, Han Yan, Tao Guo, Lei Chen, Di Yang, Yu Hu

**Affiliations:** 1 Institute of Hematology, Union Hospital, Tongji Medical College, Huazhong University of Science and Technology, Wuhan, China; 2 Deparment of Experimental Animals, Tongji Medical College, Huazhong University of Science and Technology, Wuhan, China; 3 Cancer Center, Union Hospital, Tongji Medical College, Huazhong University of Science and Technology, Wuhan, China; Faculté de médecine de Nantes, France

## Abstract

Brain-derived neurotrophic factor (BDNF) was recently identified as a factor produced by multiple myeloma (MM) cells, which may contribute to bone resorption and disease progression in MM, though the molecular mechanism of this process is not well understood. The purpose of this study was to test the effect of BDNF on bone disease and growth of MM cells both in vitro and in vivo. Co- and triple-culture systems were implemented. The in vitro results demonstrate that BDNF augmented receptor activator of nuclear factor kappa B ligand (RANKL) expression in human bone marrow stromal cells, thus contributing to osteoclast formation. To further clarify the effect of BDNF on myeloma bone disease in vivo, ARH-77 cells were stably transfected with an antisense construct to BDNF (AS-ARH) or empty vector (EV-ARH) to test their capacity to induce MM bone disease in SCID–rab mice. Mice treated with AS-ARH cells were preserved, exhibited no radiologically identifiable lytic lesions and, unlike the controls treated with EV-ARH cells, lived longer and showed reduced tumor burden. Consistently, bones harboring AS-ARH cells showed marked reductions of RANKL expression and osteoclast density compared to the controls harboring EV-ARH cells. These results provide further support for the potential osteoclastogenic effects of BDNF, which may mediate stromal–MM cell interactions to upregulate RANKL secretion, in myeloma bone diseases.

## Introduction

Multiple myeloma (MM) is a B-cell malignancy characterized by clonal expansion of plasma cells within the bone marrow and the development of destructive bone disease. The principal cellular mechanism involved in the development of myeloma bone disease is increased osteoclastic bone resorption that is not accompanied by proportional osteoblastic bone formation [1]. Bone resorption in MM is a local event that occurs adjacent to myeloma cells and is correlated with tumor burden, suggesting the capacity of myeloma cells to induce osteolytic bone destruction in the bone marrow (BM) milieu. The pathophysiology of MM-induced osteoclastogenesis involves direct production of osteoclast-activating factors (OAFs) by myeloma cells, as well as indirect interactions between myeloma cells and the bone marrow microenvironment [2]. Consistent with this theory, bone marrow stromal cells (BMSCs), one of the most pivotal cell types in the BM milieu, provide various OAFs via stroma-myeloma interactions to enhance osteoclast formation in MM. Osteoclastogenic factors involved in this process include receptor activator of nuclear factor kappa B ligand (RANKL), macrophage inflammatory protein-1α (MIP-1α), tumor necrosis factor-α (TNF-α), hepatocyte growth factor (HGF), interleukin-11 (IL-11), and stromal-derived factor-1α (SDF-1α) [3], [4], [5], [6].

RANKL, a factor mainly produced by stromal/osteoblast cells in the BM milieu, is one of the most important factors in the regulation of osteoclastogenesis [7]. RANKL directly induces osteoclast formation and inhibits osteoclast apoptosis by binding to its specific receptor (RANK) on osteoclast-lineage cells [8]. RANKL/RANK signaling is both necessary and sufficient for the complete differentiation of osteoclast precursors into mature osteoclasts in vitro [7]. Mice deficient in RANKL show decreased osteoclast activity and develop osteopetrosis, indicating the critical role of RANKL in normal osteoclast biology [9]. RANKL expression in stromal/osteoblast cells is abnormally upregulated in osteolytic bone lesions of myeloma patients [10], [11], suggesting that some factors derived from myeloma cells may increase RANKL expression in bone marrow to promote osteolytic bone disease.

In recent years, numerous studies have revealed the important role of BDNF in the pathogenesis of both neuronal and non-neuronal tumors, including multiple myeloma [12], [13], [14], [15], [16], [17], [18], [19]. The critical roles of BDNF in MM pathophysiology are evidenced by its over-expression in malignant plasma cells and myeloma cell lines, as well as its potential ability to promote the growth of MM cells in vitro [20], [21]. Recently, BDNF was identified as a potential osteoclastogenic factor in multiple myeloma, and its serum level correlated positively with that of soluble RANKL [22]. However, the mechanism by which osteoclastogenesis is promoted by BDNF in MM has not yet been clearly elucidated. Because several studies have confirmed that both BMSCs and osteoblasts express tyrosine receptor kinase B (TrkB), the high-affinity receptor of BDNF on their surface [23], [24], these findings suggest that osteoclastogenesis in myeloma may be promoted by BDNF partially through myeloma-stroma interactions that induce RANKL secretion in the BM microenvironment.

The primary purpose of this study was to clarify the capacity of MM-derived BDNF to enhance RANKL expression and osteoclast formation in a co-culture system and a novel triple-culture system. Secondly, we aimed to investigate whether silencing of BDNF in ARH-77 cells with specific short-hairpin RNA (shRNA) blocked in vivo tumorigenesis and osteoclastogenesis and prolonged survival in the SCID-rab model of myeloma bone diseases.

## Materials and Methods

### Ethics Statements

All of experiments involving human participants were approved by the ethics committee of Union Hospital, Huazhong University of Science and Technology and complied with the principles expressed in the Declaration of Helsinki. Written informed consent was obtained from all participants. All animal experiments were carried out in strict accordance with the recommendations in the Guide for the Care and Use of Laboratory Animals of the National Institutes of Health. The protocol was approved by the Committee on the Ethics of Animal Experiments of Tongji Medical College, Huazhong University of Science and Technology (Permit Number: S227). All surgery was performed under sodium pentobarbital anesthesia, and all efforts were made to minimize suffering.

### Reagents and kits

Human recombinant BDNF (PeproTech, Princeton, NJ, USA), TrkB-specific inhibitor K252-a (Sigma-Aldrich, Deisenhofen, Germany), OPG and a neutralizing antibody to BDNF (PeproTech, Princeton, NJ, USA) were obtained and reconstituted according to the manufacturers' specifications. Antihuman BDNF and antihuman RANKL antibodies were purchased from Santa Cruz Biotechnology (Santa Cruz, CA, USA). Anti-phospho ERK1/2, anti-ERK1/2, anti-phospho Akt, anti-Akt, and anti-IκB antibodies were purchased from Cell Signaling (Danvers, MA, USA). Recombinant human macrophage colony-stimulating factor (M-CSF) and RANKL were purchased from R&D Systems (Minneapolis, MN, USA). A leukocyte acid phosphatase kit for tartrate-resistant acid phosphatase (TRAP) staining was purchased from SIGMA (St. Louis, MO, USA). U0126 and LY204002 were purchased from Promega (Southampton, Hants, UK). The transwell inserts with 0.4 µm pores were obtained from Costar (Corning, NY, USA).

### Cell culture

The human MM cell lines (HMCLs) ARH77 and RPMI-8226 were purchased from the American Type Culture Collection (ATCC, Manassas, VA, USA). Multiple myeloma plasma cells (MMPCs) were obtained from 3 independent MM patients as previously described [25]. Marrow plasma samples were collected from 22 patients who gave written informed consent. Basal levels of BDNF and RANKL in these marrow plasma were measured using Human BDNF Quantikine ELISA kit and Human RANKL Quantikine ELISA kit from R&D Systems (Minneapolis, MN). Human primary BMSCs and pre-osteoclasts were prepared and identified as previously described [22], [26]. Then a series of co- and triple- culture systems (MM-BMSCs, BMSCs-preOCs, MM-BMSCs-preOCs) were implemented and treated with various conditions. Additional details are shown in the Supporting information.

### Stable transfection of shRNA/eGFP in MM cell lines by lentiviral vectors

To directly identify the biological effects of BDNF on MM, an antisense construct to BDNF (short-hairpin RNA, shRNA) was designed as previously described [26]. The sequences of the two strands were the following: 5′- CCGGCCGGCATTGGAACTCCCAGTGTTCAAGACGCACTGGGAGTTCCAATGCCTTTTTTG -3′ and 5′-AATTCAAAAAAGGCATTGGAACTCCCAGTGCGTCTTGAACACTGGGAGTTCCAATGCCGG -3′. An eGFP reporter gene was also used. ARH-77 cells were transfected with BDNF antisense shRNA/eGFP (AS-ARH) or empty-vector shRNA/eGFP (EV-ARH) by replication-incompetent lentiviral vectors. The expression of eGFP was detected by an LSR II flow cytometer (Becton Dickinson, Franklin Lakes, NJ, USA) and analyzed by BD FACSDiva software. Then, the eGFP-positive cells were sorted on a FACSDiva (Becton Dickinson, Franklin Lakes, NJ, USA). Down-regulation of BDNF protein expression was confirmed by western blotting. The detailed characteristics of AS-ARH, EV-ARH and WT- ARH cells are shown in the Supporting information.

### SCID-rab-MM mouse model and in vivo treatments

Six-week-old male NOD/SCID mice were obtained from Beijing Hua Fukang Bioscience Company, and 3-week-old New Zealand rabbits were from the Wuhan Centers for Disease Prevention & Control. Rabbits were deeply anesthetized to excise the femora and tibiae, which were then cut into two pieces, with the proximal and distal ends kept closed. Rabbit bone grafts were subcutaneously implanted into NOD/SCID mice (SCID-rab mice) as previously described [25]. Four weeks following bone implantation, AS-ARH, EV-ARH, and wild-type ARH77 (WT-ARH) cells (1.0×10^6^ cells/mouse) in 25 µl of phosphate-buffered saline (PBS) were injected directly into the implanted bone (n = 12 per group). Because ARH-77 cells release soluble human IgG, we used IgG as a quantifiable indicator of MM growth and tumor burden in SCID-rab mice. Blood samples were collected periodically, and murine sera were analyzed for human IgG by enzyme-linked immunosorbent assay (ELISA; R&D Systems). Radiographs were taken with an AXR Minishot-100 beryllium source instrument (Associated X-Ray Imaging Corp, Haverhill, MA, USA). Changes in the bone mineral density (BMD) of the rabbit bone grafts were measured by a PIXImus dual-energy x-ray absorptiometry (DEXA) device (GE Medical Systems LUNAR, Madison, WI, USA). The fluorescence intensity of SCID-rab mice was monitored periodically using the Lumazone FA 2048 system (Roper Scientific, USA), following a cutaneous shave of the tumor area. Images were analyzed using MAG Biosystems software. At the end of the experiment, mice were deeply anesthetized with pentobarbital and euthanized by cervical dislocation. Rabbit bone grafts were fixed, decalcified, and embedded for sectioning. Then, the sections were stained with Hematoxylin and eosin (H&E) staining or TRAP staining. For ELISA analysis, marrow plasma of the implanted bones was obtained by flushing the bones repeatedly with 1 ml of PBS. Then, RANKL levels in bone marrow plasma were measured. For immunohistochemistry analysis, sections were reacted with 1∶1000 diluted RANKL monoclonal antibodies and corresponding secondary antibodies.

### Real time-polymerase chain reaction

Total RNA was extracted with Trizol reagent (Invitrogen, Carlsbad, CA), and an aliquot (1 µg) of purified total RNA was subjected to reverse transcription-polymerase chain reaction using the SuperScript First-Strand Synthesis System for RT-PCR (Invitrogen). cDNAs were used as templates in real time-polymerase chain reaction with the SYBR Green RT-PCR Kit (TAKARA). DNA was amplified under the following typical cycling conditions: denaturation at 95°C for 1 min, annealing at 60°C for 30 seconds, extension at 72°C for 30 seconds. The samples were amplified for 35 cycles. GAPDH was used as an internal control. The following primers were used: for RANKL, forward primer 5′-CAA GAG GAC AGA CTC ACT TTA T -3′, reverse primer 5′–TAT CGT TGG ATC ACA GCA C-3′; for GAPDH, forward primer 5′-GAC ATC AAG AAG GTG GTG AA-3′, reverse primer 5′-TGT CAT ACC AGG AAA TGA GC-3′. Then, the PCR products were mixed with bromophenol blue-loaded buffer, electrophoretically separated in a 2% agarose gel in TAE buffer, and photographed.

### Western blotting

Cells exposed to various conditions were lysed using lysis buffer (20 mM Tris-HCl (pH 8.0), 150 mM NaCl, 2 mM EDTA, 100 mM NaF, 1% NP40, 1 µg/ml leupeptin, 1 µg/ml antipain, 1 mM PMSF), and extracted for 20 min on ice. Whole-cell extract protein (30 µg) was resolved on 10% SDS- PAGE gel and transferred to a PVDF membrane (Amersham), blotted with antibodies and then detected using the ECL Plus detection system.

### Immunofluorescence of p65 localization

To examine the translocation of nuclear factor kappa B (NF-κB), BMSCs were plated onto glass coverslips and treated with DMEM alone or DMEM containing 25 ng/ml BDNF (PeproTech) for 15, 30 or 60 min. Following fixation, NF-κB p65 localization was evaluated by incubating BMSCs with rabbit anti-human monoclonal NF-κB p65 (Santa Cruz Biotechnology) and FITC-conjugated goat anti-rabbit IgG (Invitrogen). Coverslips were then treated with 4,6-diamidino-2-phenylindole (DAPI) (Invitrogen).

### Statistical analysis

In vitro experiments for all assays were performed in triplicate, and the results are reported as the means ± SEMs. Statistical analysis of the treatment groups compared with their respective control groups was performed using ANOVA, and *P*<0.05 was considered statistically significant. The significance of differences in overall survival between each group was analyzed by Kaplan–Meier curves and log-rank tests.

## Results

### BDNF increases RANKL mRNA and protein levels in human BMSCs

**Figure 1:**
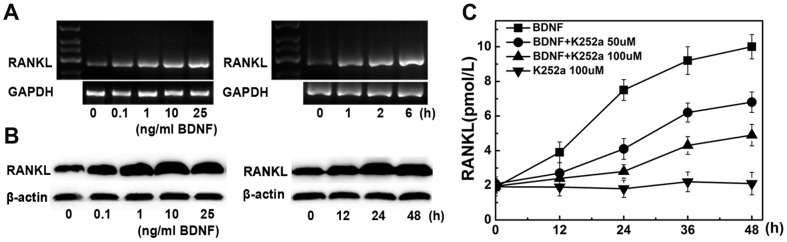
Effects of BDNF on RANKL expression in human bone marrow stromal cells (BMSCs). (A) The dose and time responses to BDNF of RANKL mRNA expression in BMSCs. PCR analysis indicates that BDNF markedly increases RANKL mRNA expression in human BMSCs (B) Western blot analysis shows that BDNF increased RANKL protein expression by human BMSCs in a dose- and time-dependent manner. (C) K252a diminishes BDNF-mediated RANKL secretion in human BMSCs. Stimulation with 25 ng/ml BDNF was performed in the absence or presence of K252a pretreatment (50, 100 nM; 60 min) in BMSCs. K252a without BDNF was used for control stimulation. RANKL levels in supernatants at different time points (0, 12, 24, 48 h) were detected by ELISA.

Human BMSCs were isolated from healthy donors and stimulated by BDNF. As shown in Figure 1A, stimulation of BMSCs with BDNF promoted RANKL mRNA expression in a dose- and time-dependent manner. RANKL mRNA began to increase 1 h after exposure to 25 ng/ml BDNF; the increase became notable at 2 h and persisted for several hours. Similar results were obtained for RANKL protein (Figure 1B): RANKL expression distinctly increased in BMSCs treated with 25 ng/ml BDNF for 48 h. In addition, K252a, a specific Trk family inhibitor, was added to BMSCs at concentrations of 10, 50, and 100 nM. As shown in Figure 1C, K252a (100 nM for 48 h) significantly diminished RANKL secretion in the supernatants of BMSCs compared to PBS-treated controls (*P*<0.01).

### BDNF induces RANKL expression in BMSCs through ERK and AKT signaling pathways, not NF-κB

**Figure 2:**
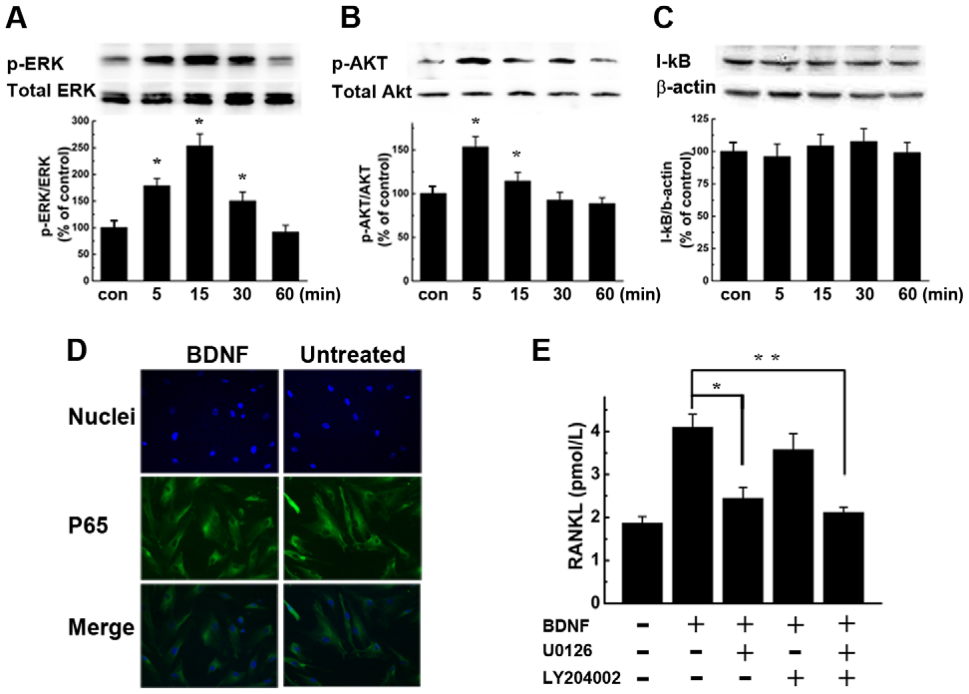
BDNF induces ERK1/2 and AKT phosphorylation in BMSCs, but has no effect on NF-κB activation. (A, B, C) BMSCs were cultured in the presence of 25 ng/ml BDNF for 5, 15, 30, or 60 min. The expression of p-ERK1/2 (A) and p-Akt (B) began to increase at 5 min. However, no obvious change in the I-κB (C) level was detected by 60 min after BDNF stimulation. The results are representative of three independent experiments. Statistical analysis was conducted by ANOVA, * *P*<0.01 versus controls. (D) BDNF does not induce translocation of NF-κB p65 to the nucleus in BMSCs. BMSCs were treated with BDNF for 5, 15, 30 or 60 minutes, then fixed and probed with antibodies specific for NF-κB p65. Magnification ×200. Representative images from 15-min exposures to stimuli are exhibited. (E) MEK1/2 inhibitor U0126 and PI3K inhibitor LY204002 blunted the increase of RANKL protein secretion induced by BDNF. The results represent the means ± SEMs for all assays (performed in triplicate) (BDNF+U0126 vs. BDNF, * *P*<0.05; BDNF+LY204002 vs. BDNF, *P* = 0.069; BDNF+U0126+LY204002 vs. BDNF, ** *P*<0.05).

To identify the downstream signaling molecules of the receptor activated by BDNF in BMSCs, we analyzed the phosphorylation of MEK/ERK and PI3K/AKT by western blot. Cells were stimulated with 25 ng/ml BDNF for different time periods. As shown in Figure 2A, the phosphorylation of ERK1/2 was detected as early as 5 min, with a peak signal at 15 minutes and a diminished signal at 30 minutes. As shown in Figure 2B, the phosphorylation of AKT showed a peak signal at 5 min, which gradually attenuated but still existed at 15 min. The total levels of these proteins remained unchanged. However, no obvious change in phosphorylation of IκB was detected up to 60 min (Figure 2C). To confirm the lack of NF-κB activation by BDNF stimulation, BMSCs were treated with 25 ng/ml BDNF for different time periods and analyzed by immunofluorescence for nuclear translocation of NF-κB p65. Consistently, no obvious nuclear translocation of the NF-κB p65 subunit was noted when compared to the untreated control (Figure 2D). To further investigate the roles of ERK1/2 and AKT signaling in the BDNF induction of RANKL production, BMSCs were pre-incubated with MEK/ERK inhibitor U0126 and or AKT inhibitor LY204002 for 1 hour and exposed to 25 ng/ml of BDNF for 24 hours. As shown in Figure 2E, BMSCs pre-treated with U0126 showed a 49.75% decrease in BDNF-stimulated RANKL secretion compared with BDNF stimulation alone (*P*<0.05). Treatment of BMSCs with LY204002 partially inhibited BDNF-stimulated RANKL secretion but with no statistical significance (P = 0.069). These findings indicate that MEK/ERK are the main downstream mediators of BDNF-induced RANKL secretion in BMSCs.

### MM-derived BDNF increases RANKL expression and osteoclast formation in co- and triple-culture systems

**Figure 3:**
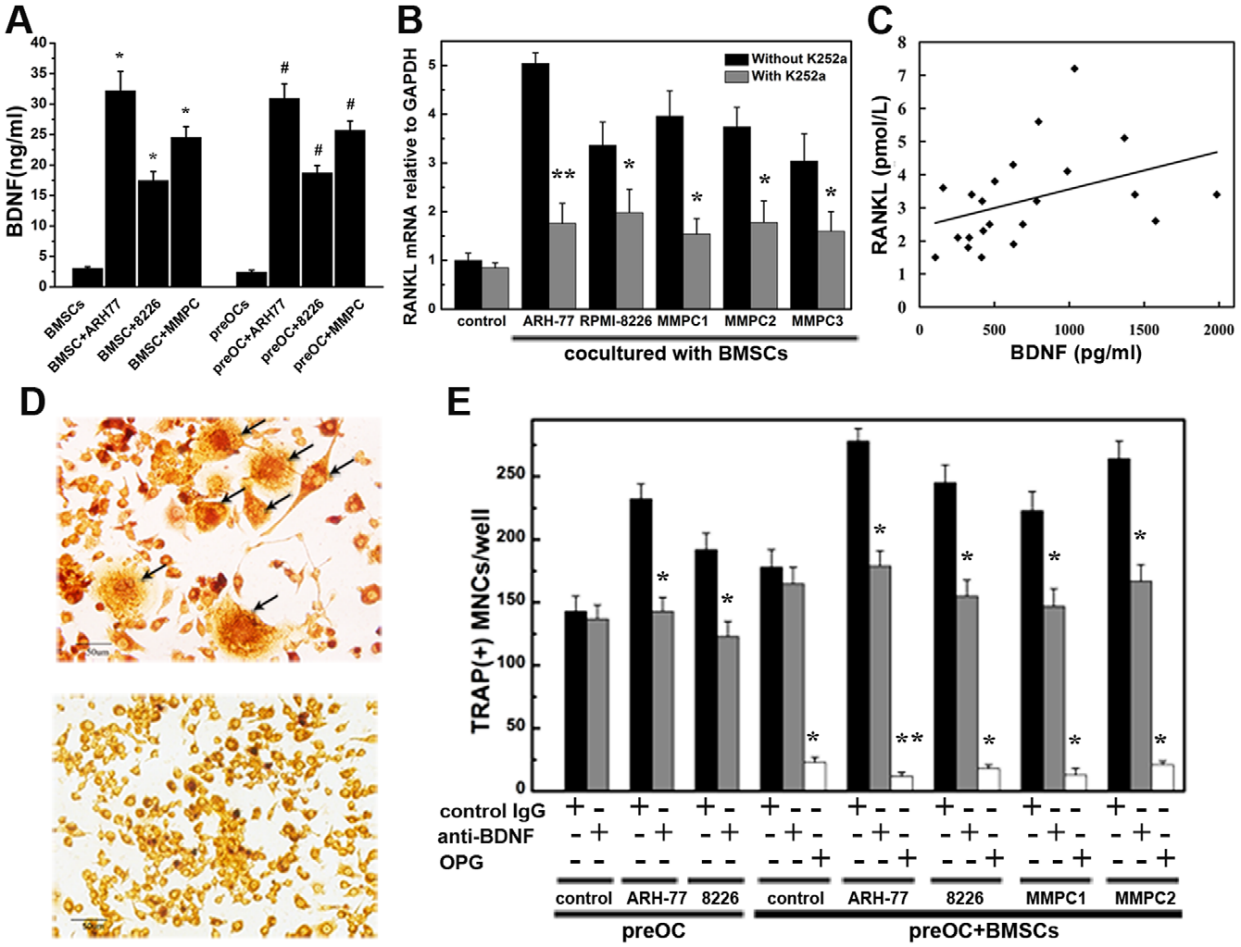
MM-derived BDNF promotes RANKL expression in co-culture systems and increases osteoclast formation in triple-culture systems. (A) Soluble BDNF levels in culture medium measured by ELISA (ng/ml). BMSCs or preOCs were cultured alone or cocultured with MM cell (ARH-77, RPMI8226, MMPCs) using the transwell systems we described in methods before. After 48 hours, the supernatants were collected, and BDNF concentrations were analyzed with an ELISA kit. Each experiment was done in triplicate. The mean levels of BDNF secretion in cocultures of both MM-BMSC and MM-preOC were significantly higher than in BMSCs or preOCs alone (* *P*<0.01 compared to BMSCs cultured alone; # *P*<0.01 compared to preOCs cultured alone). (B) RANKL mRNA increased when BMSCs were cocultured with MM cells. These effects were abolished by K252a. The results represent the means ± SEMs for all assays (performed in triplicate). * *P*<0.05, ** *P*<0.01 compared to the control cultured without K252a. Control means the basal level of RANKL mRNA when BMSCs were cultured alone. (C) Correlation between BDNF levels in bone marrow plasma from 22 patients and marrow plasma-induced RANKL secretion by BMSCs (correlation coefficient = 0.35, *P*<0.01). (D) Representative images of TRAP-positive multinucleated osteoclast-like cells from human peripheral blood mononuclear cell cultures (labeled by black arrows) and the negative control. Magnification ×200. (E) Osteoclast precursors were cocultured with MM cells or triple-cultured with both BMSCs and MM cells. The enhancement of osteoclast formation in co- and triple-cultures was reversed by BDNF-neutralizing antibody (gray lanes) and was nearly completely abolished by OPG (white lanes). Goat IgG was used as a control (black lanes). * *P*<0.05, ** *P*<0.01 compared to the control.

First, we detected the basal levels of soluble BDNF in cultures of MM cells alone with ELISA analysis, and consistent with previous studies [19], [27], our results demonstrated the secretion of BDNF by MM cells (28.75±1.21 ng/ml for ARH-77 cells, 15.31±0.78 ng/ml for RPMI-8226 cells and 21.52±1.33 ng/ml for MMPCs). Then a series of co-cultures was performed in a non-contact transwell system to investigate the potential effect of MM-derived BDNF on RANKL expression in human BMSCs. BDNF secretion levels significantly increased in co-culture systems of both MM-BMSCs and MM-preOCs compared to BMSCs or preOCs cultured alone (Figure 3A). As shown in Figure 3B, RANKL mRNA levels were significantly higher than control (BMSCs cultured alone) when BMSCs were co-cultured with ARH-77, RPMI-8226, or MMPCs from 3 independent myeloma patients. The stimulatory effect was more pronounced in ARH-77 than RPMI-8226 cells. Pretreatment with K252a completely abolished the enhancement of RANKL expression induced by MM cells. Bone marrow plasma from 22 MM patients with or without bone lesions was added to BMSCs culture systems. Basal levels of BDNF and RANKL from these bone marrow plasma were measured by ELISA. Results demonstrated a positive correlation between bone marrow plasma BDNF levels and bone marrow plasma RANKL levels (*r* = 0.63, *p*<0.01). Then we investigate the relationship of BDNF levels in patient bone marrow plasma and marrow plasma-induced RANKL secretion by BMSCs. As shown in Figure 3C, the relative level of RANKL secretion in BMSCs induced by MM bone marrow correlated positively with BDNF level in each marrow plasma sample (correlation coefficient = 0.35). Bone marrow plasma from MM patients with higher levels of BDNF had a greater ability to induce RANKL secretion in BMSCs. We next investigated the effects of BDNF on osteoclast differentiation in our co- and triple-culture systems. Representative images of TRAP-positive multinucleated osteoclast-like cells (OCLs) are shown in Figure 3D. As shown in Figure 3E, a marked increase of osteoclast number was detected when pre-OCs were co-cultured with MM cells or triple-cultured with human BMSCs and MM cells. Importantly, these effects were partially reversed by neutralizing antibody to BDNF and completely abolished by OPG, indicating the potential effects of MM-derived BDNF on osteoclast formation.

### Stable knockdown of BDNF in ARH77 cells restrains bone destruction in SCID-rab model

**Figure 4:**
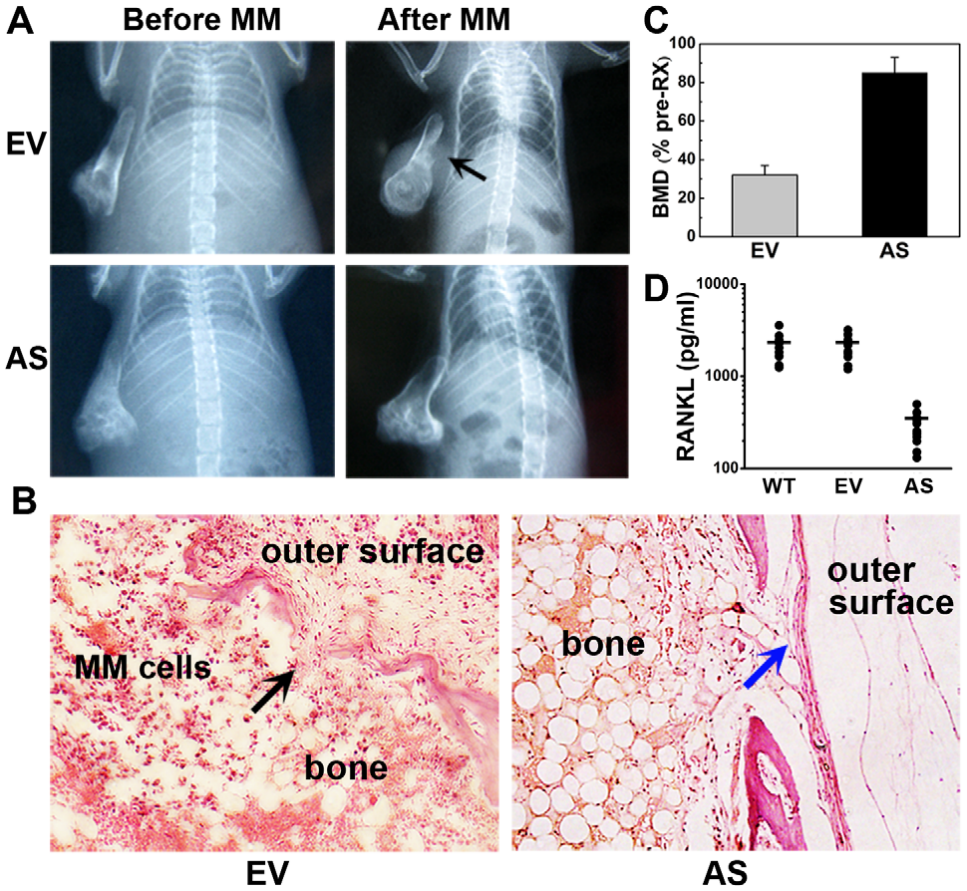
Antisense inhibition of BDNF in ARH77 cells prevents tumor-induced osteolytic lesions in bone implants. (A) Representative X-ray radiographs of the implanted myelomatous bones in each group, before cell engraftment (Before MM) and at the end of the experiment (After MM). Bones engrafted with EV-ARH cells were severely damaged, and tumors grew on the outer surface of the implanted bone (black arrow). However, no obvious osteolytic destruction was detected in bones harboring AS-ARH cells. (B) H&E staining of bone sections. Bones in the EV-ARH group were severely resorbed. Tumors damaged normal bone structures and infiltrated to the outer surface of the implanted bones (black arrow). In contrast, bones harboring AS-ARH cells had relatively normal structures and continuous cortexes (blue arrow). Magnification ×100. (C) Changes in the BMD of implanted bones. Error bars represent SEMs, *P*<0.01. (D) Changes in the level of soluble RANKL in rabbit bone implants. The mean levels of RANKL protein expression in WT-, EV-, and AS- group were 2205.9, 2130.5, and 286.3 pg/ml, respectively. RANKL levels in AS- group were significantly reduced when compared to EV- group (*P*<0.01).

AS-, EV-, and WT-ARH cells were injected into rabbit bone grafts to investigate the effect of BDNF on bone destruction in vivo. Radiographic analysis of the engrafted bones (Figure 4A) showed that as predicted, bones harboring AS-ARH cells, which expressed low levels of endogenous human BDNF, had a lower incidence of osteolytic lesions compared to the EV-ARH group (n = 12 per group, *P* = 0.02). Bones in the EV-ARH group were severely resorbed, and in some representative cases (Figure 4B), tumors broke through the bone cortex and extended to the outer surface of the implanted bones. In contrast, bones harboring AS-ARH cells had relatively normal bone structures and continuous cortexes, and tumor cells were restricted to bones. Changes in BMD in the implanted rabbit bones were analyzed to better quantify the osteolytic burden. As shown in Figure 4C, the osteolytic burden of rabbit bones from the AS group was significantly lower than that from the EV-ARH group. The BMD of bones injected with EV-ARH cells was reduced by 68%±5% compared to pretreatment BMD, whereas in the AS-ARH group, BMD was decreased by 15%±8% (EV-ARH vs. AS-ARH, n = 12 per group, *P*<0.05). WT-ARH cells (n = 12) were not used for this because of the similarity of their clinical characteristics to those of the EV-ARH group. Then we detected RANKL levels in our in vivo model with ELISA kits. As shown in figure 4D, bones harboring AS-ARH cells, which expressed low levels of endogenous human BDNF, had a significantly reduced level of RANKL compared to the EV-ARH group (*P*<0.01).

### Stable knockdown of BDNF attenuates RANKL expression and osteoclastogenesis in myelomatous bones

**Figure 5:**
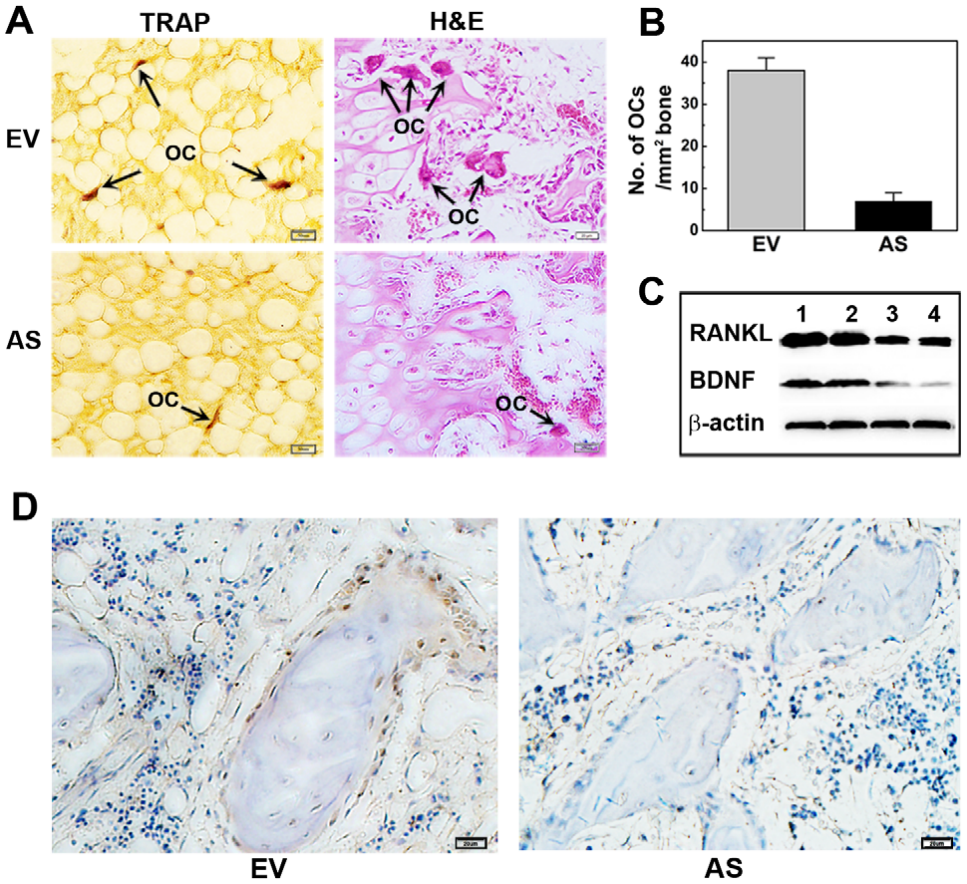
Antisense inhibition of BDNF inhibits osteoclast formation and RANKL expression in myelomatous bones. (A) TRAP and H&E staining of the myelomatous bones. Representative osteoclasts are labeled with arrows. Magnification ×200. (B) Quantitative analysis of osteoclasts on trabecular bone surface. Data represent the means ± SEMs of five separate high-power fields (HPFs). (C) Bone engrafts were fetched and cells in bones were obtained by flushing the bones repeatedly with 1 ml of PBS. Expression levels of RANKL and BDNF were measured by western blot Results indicate that RANKL and BDNF protein level in AS-ARH-infiltrated bones (lanes 3, 4) were much lower than those in EV-ARH group (lanes 1, 2). (D) Immunohistochemistry analysis indicates that the number of RANKL-positive cells in bone sections from the AS-ARH group was markedly decreased compared to the EV-ARH group .Magnification ×400.

Myeloma osteolytic lesions are thought to result from increased osteoclastogenesis induced by the MM-related microenvironment. As shown in Figure 5A, TRAP-positive multinucleated OCLs in rabbit bone implants from the AS-ARH group were less numerous than those from the EV-ARH group. Histomorphometric analysis was utilized to better quantify the osteoclast activity. As shown in Figure 5B, OCL number per square millimeter of trabecular bone surface in the AS-ARH group was significantly reduced in comparison with the EV-ARH group. To investigate the molecular mechanism by which antisense inhibition of BDNF reduces osteoclast formation in SCID-rab mice, RANKL, the final and critical osteoclastogenic factor primarily expressed by stromal/osteoblast cells, was detected by western blot analysis. Result in Figure 5C showed significantly lower expression of RANKL and BDNF in AS-ARH-infiltrated bone. Consistent with this result, the number of RANKL-positive cells in bone sections from the AS-ARH group was markedly decreased compared to the EV-ARH group when detected by immunohistochemistry analysis (Figure 5D). Together, these findings indicate that silencing BDNF activity in ARH77 cells decreased RANKL expression in the bone marrow microenvironment and resulted in attenuated osteoclastogenesis in vivo.

### Stable knockdown of BDNF inhibits tumor growth and prolongs survival in SCID-rab mice

**Figure 6:**
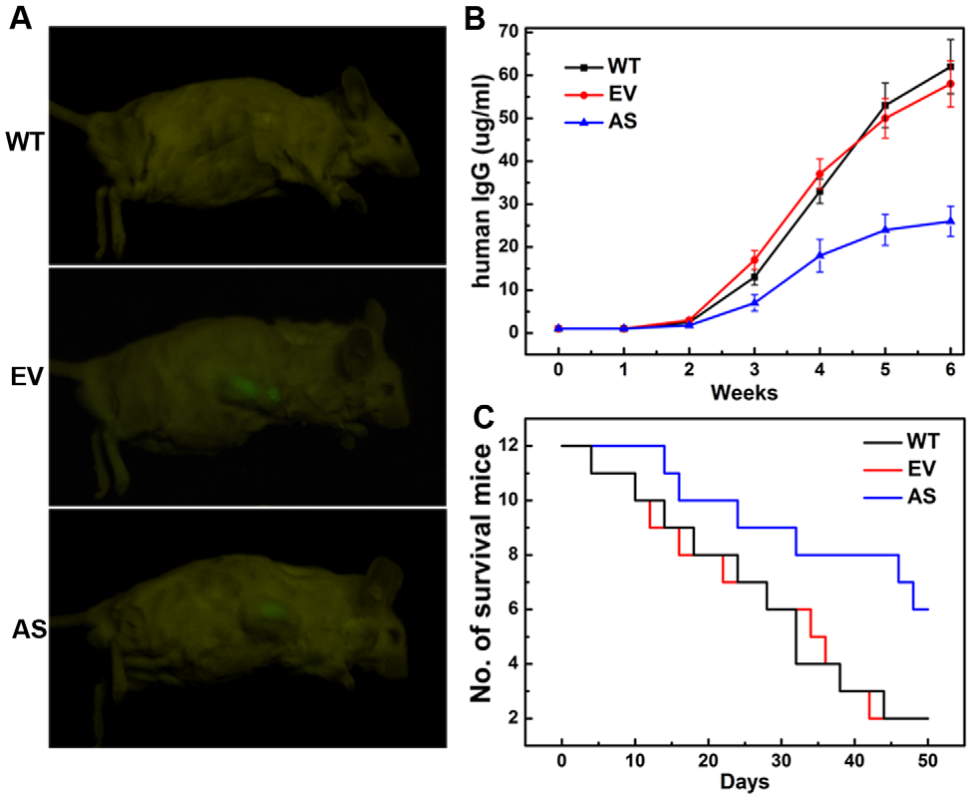
Stable knockdown of BDNF activity inhibits tumor growth and prolongs overall survival in vivo. (A) Representative fluorescence images of SCID-rab mice harboring the myeloma xenograft tumors. Mice harboring AS-ARH cells suffered lower tumor burden than animals harboring EV-ARH cells. (B) Circulating human IgG levels in SCID-rab mice were detected by ELISA. Human IgG levels began to show a difference 2 weeks after ARH inoculation, and the difference became increasingly marked. Circulating human IgG levels in the AS-ARH group was significantly lower than the EV-ARH and WT-ARH group at 6 weeks (*P*<0.05). (C) The effects of BDNF knockdown on overall survival were analyzed by Kaplan–Meier curves and long-rank tests.

SCID-rab mice were monitored periodically by real-time fluorescence imaging as an indicator of in vivo MM tumor growth. As shown in Figure 6A, the fluorescence intensity in the AS-ARH group was much weaker than those in the EV-ARH groups. Because ARH cells produce human IgG, tumor burden was also assessed based on changes in human immunoglobulin light chains in blood serum. AS-, EV- and WT-ARH cells were first confirmed to secrete equal amounts of human IgG (data not shown), and then all mice were injected with the same number of cells. Three weeks after inoculation, circulating hIgG in blood serum from the AS-ARH group (n = 12) was significantly lower than in the EV-ARH (n = 12) and WT-ARH (n = 12) groups (*P*<0.05 for both) (Figure 6B). Moreover, Kaplan–Meier curves and log-rank analysis showed significantly prolonged overall survival of the mice in the AS-ARH group compared with EV-ARH and WT-ARH (*P*<0.05 for both) (Figure 6C).

## Discussion

The current study demonstrates that MM-derived BDNF promotes osteoclast formation via upregulation of RANKL expression in vitro, and antisense inhibition of BDNF in ARH-77 cells blocks osteolytic bone destruction and tumor growth in SCID-rab mice. These results suggest that BDNF may be an important factor in the bone-destructive process and disease progression in MM.

In this study, we found that BDNF promoted the secretion of RANKL in human BMSCs to further amplify osteoclast formation in vitro. Co- and triple-culture systems mimicking the marrow microenvironment revealed that MM-induced osteoclast formation was partially blocked by BDNF-neutralizing antibody and was nearly completely blocked by OPG, revealing that the significant effects of MM-derived BDNF on osteoclastogenesis may occur through MM-stromal interactions that upregulate RANKL expression. RANKL is the dominant and final mediator of osteoclast formation. An abnormally increased level of RANKL was found in the MM-related bone marrow microenvironment, which contributes to osteolytic bone destruction and disease progression [10], [11]. RANKL expression is promoted by several factors produced or induced by MM cells, such as TNF-α, MIP-1α, IL-1, IL-6, and PTH [4]. Combined with our previous observation that serum RANKL level in MM patients correlated positively with that of soluble BDNF, these results suggest that BDNF may be an important contributing factor to the RANKL pool in bone, thus promoting osteoclast formation and bone destruction in MM. RANKL is produced by not only BMSCs and osteoblasts but also MM cells themselves in the BM milieu [28], [29]. Because the BDNF/TrkB signaling pathway is involved in many biological activities of MM cells, these results raise the possibility that BDNF may also influence RANKL expression in MM cells through an autocrine pathway, which needs to be investigated in the future.

BDNF stimulation activates the MAPK and PI3K/AKT pathways in neurons and neural stem cells [30], [31], [32], [33], [34]. Moreover, the MAPK and PI3K/AKT signaling pathways have been implicated in regulating RANKL secretion in bone marrow stromal/osteoblastic cells [3], [35], [36]. These observations are in agreement with the results of our study, which showed the MEK/ERK and PI3K/AKT pathways are involved in the promotion of RANKL expression. NGF activates the NF-κB pathway in rat pheochromocytoma PC12 cells [37]. However, no obvious phosphorylation of IκB or nuclear translocation of NF-κB p65 was detected in this study. Further investigation is required to characterize the relationship between NF-κB activation and RANKL up-regulation in BMSCs in detail.

The stable knockdown of BDNF in ARH77 cells decreased bone destruction in SCID-rab mice. There are several possible explanations for these results. The first is that blocking BDNF activity suppresses RANKL expression by BMSCs in the BM milieu, as further confirmed by ELISA, western bolt, and immunohistochemical analysis in the present study. Second, antisense inhibition of BDNF in MM cells blocks VEGF secretion by BMSCs [26]. VEGF, an angiogenic factor, also participates in a vicious cycle between angiogenesis and osteoclastogenesis in MM [38]. Third, BDNF promotes survival and blocks apoptosis in multiple myeloma cells [21], [39]. MM cells play an important role in promoting osteoclastogenesis both by directly contacting osteoclasts and by indirectly producing osteoclast-activating factors, such as MIP-1α [40], to induce osteoclast formation. Therefore, inhibition of BDNF activity may block MM survival, resulting in an attenuated osteoclastic effect by MM cells.

Antisense inhibition of BDNF activity in ARH cells also decreased tumor burden and prolonged survival in SCID-rab mice. One explanation for this result is that BDNF is required for the growth of myeloma cells. The direct effect of BDNF to improve MM growth and migration has been demonstrated before [21], [39]. Our previous study also demonstrated that inhibition of BDNF in RPMI8226 could decrease tumor burden in SCID mice subcutaneously injected with MM cells [26]. Thus it is possible that antisense inhibition of BDNF abolished the direct promotion of BDNF on tumor growth. Moreover, BDNF may induce factors produced by marrow stromal cells to enhance the growth and survival of myeloma cells in the marrow. In support of this possibility are the results of Rezaee et al. [41], who showed that neurotrophins, including BDNF, regulate the expression of IL-6, a crucial factor in MM survival. Other factors induced by this process need to be identified by further research. Finally, osteoclasts produce a variety of factors to support myeloma cell growth, thereby creating a vicious cycle between OCs and MM cells [42]. Therefore, we suppose that silencing of MM-derived BDNF also attenuates RANKL expression and decreases OC activity in the BM milieu, resulting in blockage of this vicious cycle, which could promote tumor growth in MM.

Taken together, our results demonstrate that myeloma-derived BDNF can promote RANKL secretion by bone marrow stromal cells in the BM milieu through ERK pathway, and that antisense inhibition of endogenous BDNF in MM cells inhibits both osteoclastogenesis and tumor growth in vivo. These findings suggest targeting BDNF as a new therapeutic strategy to improve outcome of MM patients.

## Supporting Information

Text S1 **Methods about cell identification and co-/triple- culture system construction.** Human primary BMSCs were prepared and identified as follows. Basically, anti-coagulated BM samples were obtained from aspirates of healthy donors after the provision of informed consent. Bone marrow mononucleated cells (2×10^6^ cells/ml) were cultured in low-sugar DMEM supplemented with 10% FBS and antibiotics. Half of the medium was replaced every 4–6 days, and adherent cells were allowed to reach 80% confluence before they were sub-cultured with trypsin-EDTA. The adherent BMSCs expressed CD105, CD44, CD13 and CD90 but not CD45, CD14 or CD34 by flow cytometry analysis. Human primary osteoclasts (OCs) were prepared and identified as follows. Briefly, peripheral blood mononuclear cells from healthy donors were cultured at 2.5×10^6^ cells/ml in α-MEM supplemented with 10% FBS, antibiotics and M-CSF (25 ng/mL). After 12–24 hours, the cultures were washed gently with fresh medium to detach and remove non-adherent cells. The remaining adherent cells were considered osteoclast precursors (pre-OCs). Pre-OCs were incubated in co- and triple-culture under various conditions as follows. After culturing for 14 days, TRAP staining was performed according to the manufacturer's instructions. TRAP-positive multinucleated cells (MNCs) were counted as the differentiation effect and compared between each group. In co-culture systems, human BMSCs in passage 3–5 were detached and resuspended in low-sugar DMEM and then replated in the lower chambers of 12-well transwell inserts with 0.4-µm pores. As shown in Figure S1A, MM cell lines or MMPCs were plated in the upper chambers of the inserts with RPMI1640 medium. For some experiments, bone marrow plasma from the 22 MM patients was directly added to the upper chambers. After culturing for 24 hours, the supernatants were analyzed for RANKL protein levels by an ELISA kit (R & D Systems), and BMSCs were collected and analyzed for RANKL mRNA levels by RT-PCR. We designed a triple-culture system also using transwell inserts with 0.4-µm pores. In this system, pre-OCs were prepared as previously described in the lower chambers of a 12-well plate. Then, human BMSCs in passage 3–5 were cultured on the backside of the insert membranes, while HMCLs were incubated thereafter in the upper chambers of the inserts, as shown in Figure S1B. All triple-culture systems were incubated in α-MEM supplemented with 10% FBS, M-CSF (25 ng/mL), RANKL (50 ng/mL) and antibiotics. Culture media were changed every 3–4 days, and TRAP staining was performed on day 14.
(DOC)

Figure S1 **Co- and triple-culture systems for investigating the effects of MM-derived BDNF on RANKL expression and osteoclast formation.**
(TIF)

Figure S2 **ARH-77 cells stably transfected with BDNF shRNA or control shRNA combined with eGFP reporter gene by lentiviral vectors.**
(TIF)
