# Supplementary material for: Inhibition of BDNF in Multiple Myeloma Blocks Osteoclastogenesis via Down-Regulated Stroma-Derived RANKL Expression Both In Vitro and In Vivo
Source: PLoS One. 2012 Oct 15;7(10):e46287. doi: 10.1371/journal.pone.0046287 (PMC3471864; doi:10.1371/journal.pone.0046287)
Supplement: Text S1 — Methods about cell identification and co-/triple- culture system construction. Human primary BMSCs were prepared and identified as follows. Basically, anti-coagulated BM samples were obtained from aspirates of healthy donors after the provision of informed consent. Bone marrow mononucleated cells (2×10^6^ cells/ml) were cultured in low-sugar DMEM supplemented with 10% FBS and antibiotics. Half of the medium was replaced every 4–6 days, and adherent cells were allowed to reach 80% confluence before they were sub-cultured with trypsin-EDTA. The adherent BMSCs expressed CD105, CD44, CD13 and CD90 but not CD45, CD14 or CD34 by flow cytometry analysis. Human primary osteoclasts (OCs) were prepared and identified as follows. Briefly, peripheral blood mononuclear cells from healthy donors were cultured at 2.5×10^6^ cells/ml in α-MEM supplemented with 10% FBS, antibiotics and M-CSF (25 ng/mL). After 12–24 hours, the cultures were washed gently with fresh medium to detach and remove non-adherent cells. The remaining adherent cells were considered osteoclast precursors (pre-OCs). Pre-OCs were incubated in co- and triple-culture under various conditions as follows. After culturing for 14 days, TRAP staining was performed according to the manufacturer's instructions. TRAP-positive multinucleated cells (MNCs) were counted as the differentiation effect and compared between each group. In co-culture systems, human BMSCs in passage 3–5 were detached and resuspended in low-sugar DMEM and then replated in the lower chambers of 12-well transwell inserts with 0.4-µm pores. As shown in Figure S1A, MM cell lines or MMPCs were plated in the upper chambers of the inserts with RPMI1640 medium. For some experiments, bone marrow plasma from the 22 MM patients was directly added to the upper chambers. After culturing for 24 hours, the supernatants were analyzed for RANKL protein levels by an ELISA kit (R & D Systems), and BMSCs were collected and analyzed for RANKL mRNA levels by RT-PCR. We design [file pone.0046287.s001.doc]

**Text S1. Methods about cell identification and co-/triple- culture system construction.**

Human primary BMSCs were prepared and identified as follows. Basically, anti-coagulated BM samples were obtained from aspirates of healthy donors after the provision of informed consent. Bone marrow mononucleated cells (2 × 10^6^ cells/ml) were cultured in low-sugar DMEM supplemented with 10% FBS and antibiotics. Half of the medium was replaced every 4–6 days, and adherent cells were allowed to reach 80% confluence before they were sub-cultured with trypsin-EDTA. The adherent BMSCs expressed CD105, CD44, CD13 and CD90 but not CD45, CD14 or CD34 by flow cytometry analysis.

Human primary osteoclasts (OCs) were prepared and identified as follows. Briefly, peripheral blood mononuclear cells from healthy donors were cultured at 2.5 × 10^6^ cells/ml in α-MEM supplemented with 10% FBS, antibiotics and M-CSF (25 ng/mL). After 12-24 hours, the cultures were washed gently with fresh medium to detach and remove non-adherent cells. The remaining adherent cells were considered osteoclast precursors (pre-OCs). Pre-OCs were incubated in co- and triple-culture under various conditions as follows. After culturing for 14 days, TRAP staining was performed according to the manufacturer’s instructions. TRAP-positive multinucleated cells (MNCs) were counted as the differentiation effect and compared between each group.

In co-culture systems, human BMSCs in passage 3-5 were detached and resuspended in low-sugar DMEM and then replated in the lower chambers of 12-well transwell inserts with 0.4-μm pores. As shown in Figure S1A, MM cell lines or MMPCs were plated in the upper chambers of the inserts with RPMI1640 medium. For some experiments, bone marrow plasma from the 22 MM patients was directly added to the upper chambers. After culturing for 24 hours, the supernatants were analyzed for RANKL protein levels by an ELISA kit (R & D Systems), and BMSCs were collected and analyzed for RANKL mRNA levels by RT-PCR.

We designed a triple-culture system also using transwell inserts with 0.4-μm pores. In this system, pre-OCs were prepared as previously described in the lower chambers of a 12-well plate. Then, human BMSCs in passage 3-5 were cultured on the backside of the insert membranes, while HMCLs were incubated thereafter in the upper chambers of the inserts, as shown in Figure S1B. All triple-culture systems were incubated in α-MEM supplemented with 10% FBS, M-CSF (25 ng/mL), RANKL (50 ng/mL) and antibiotics. Culture media were changed every 3-4 days, and TRAP staining was performed on day 14.
