# Supplementary figures and images for: Inhibition of BDNF in Multiple Myeloma Blocks Osteoclastogenesis via Down-Regulated Stroma-Derived RANKL Expression Both In Vitro and In Vivo
Source: PLoS One. 2012 Oct 15;7(10):e46287. doi: 10.1371/journal.pone.0046287 (PMC3471864; doi:10.1371/journal.pone.0046287)

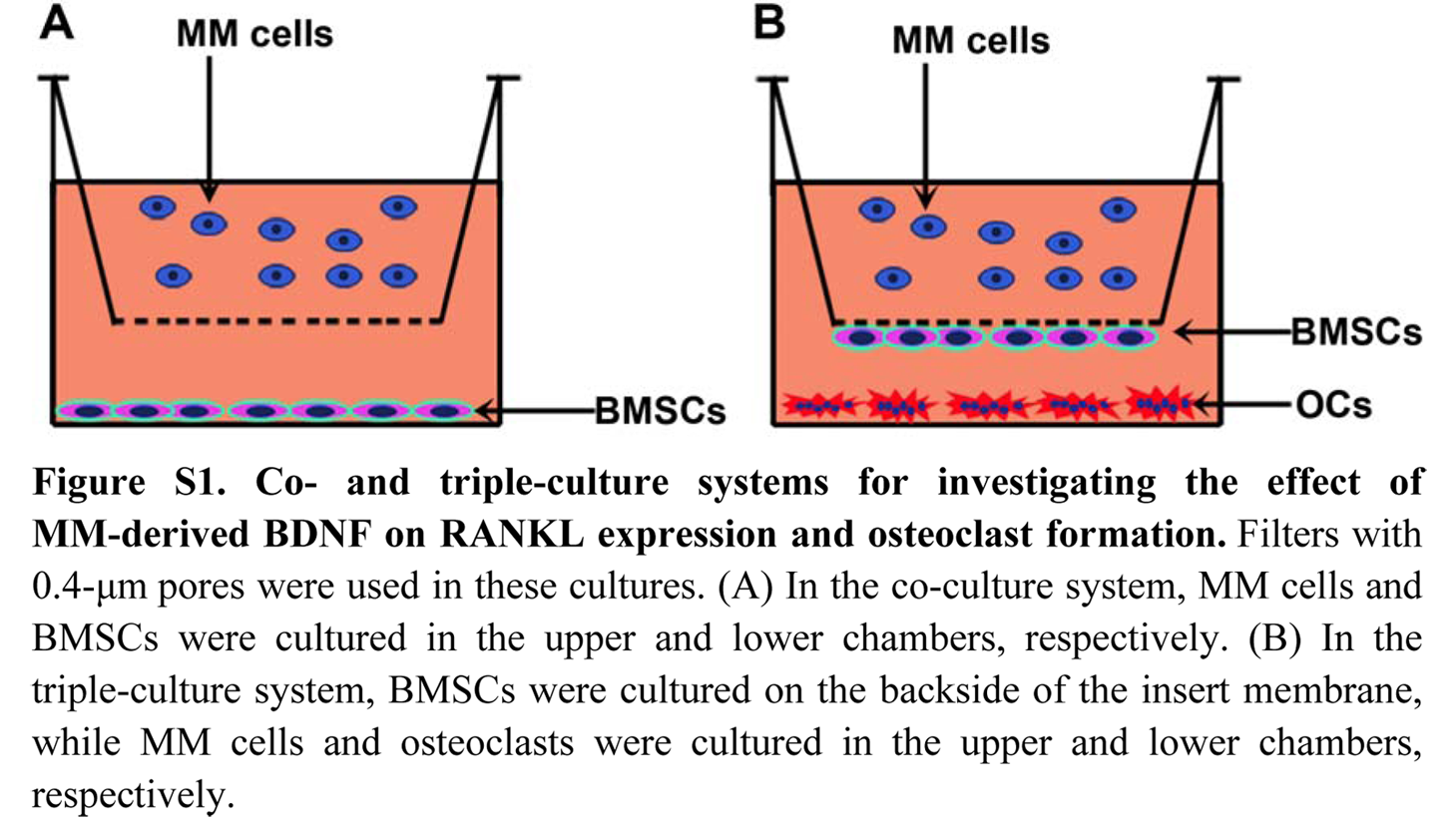

Supplement: Figure S1 — Co- and triple-culture systems for investigating the effects of MM-derived BDNF on RANKL expression and osteoclast formation. (TIF) [file pone.0046287.s002.tif]

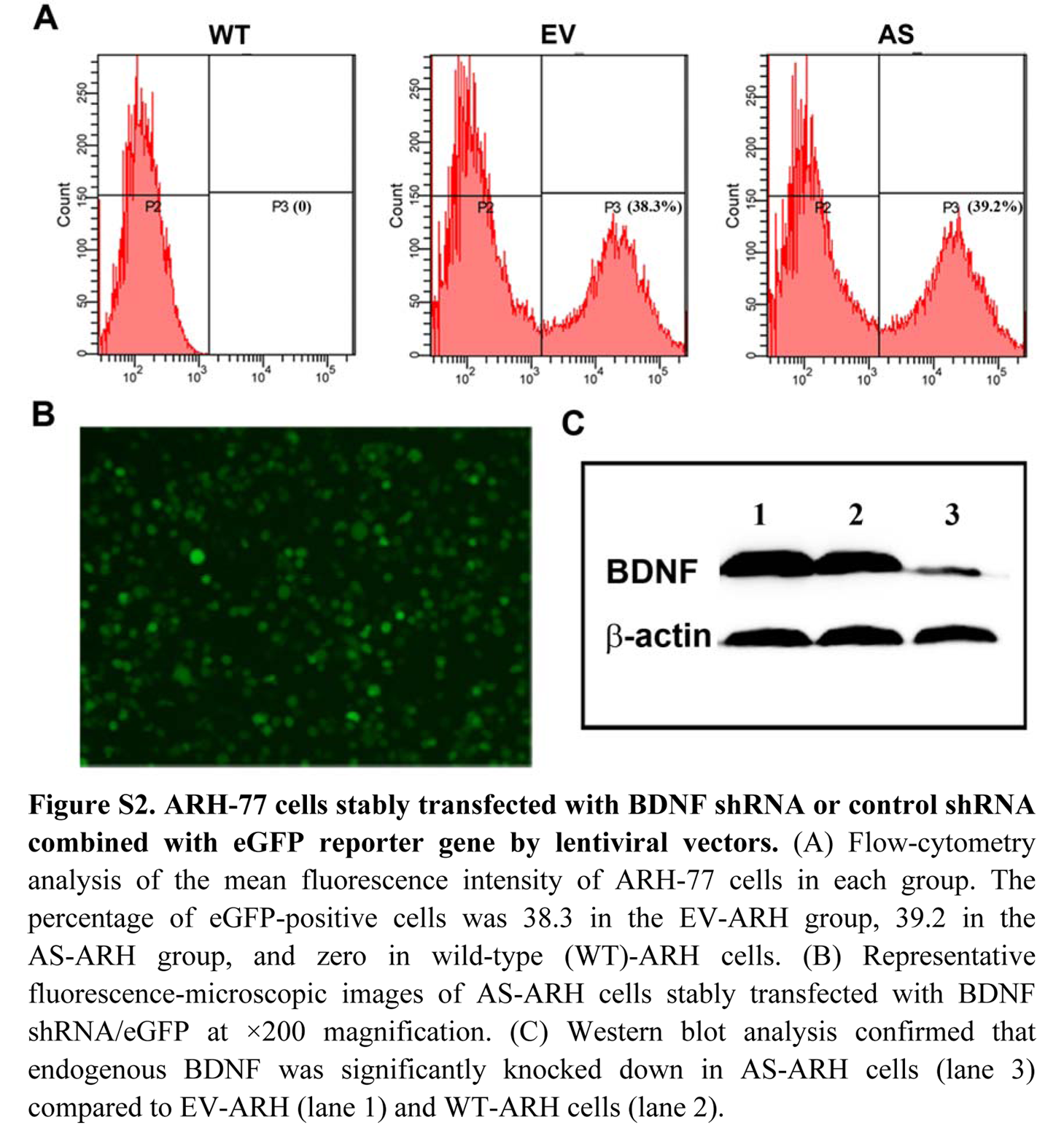

Supplement: Figure S2 — ARH-77 cells stably transfected with BDNF shRNA or control shRNA combined with eGFP reporter gene by lentiviral vectors. (TIF) [file pone.0046287.s003.tif]
